# Efficacy and HIV drug resistance profile of second-line ART among patients having received long-term first-line regimens in rural China

**DOI:** 10.1038/srep14823

**Published:** 2015-10-08

**Authors:** Jing Wang, Zhe Wang, Jia Liu, Yanchao Yue, Shimei Yang, Huimin Huang, Cui He, Lingjie Liao, Hui Xing, Yuhua Ruan, Yiming Shao

**Affiliations:** 1 State Key Laboratory of Infectious Disease Prevention and Control, National Center for AIDS/STD Control and Prevention, Chinese Center for Disease Control and Prevention, Collaborative Innovation Center for Diagnosis and Treatment of Infectious Diseases, Beijing, China; 2 Guangxi Center for Disease Control and Prevention, Nanning, China; 3 Henan Center for Disease Control and Prevention, Zhengzhou, China; 4 Xincai Center for Disease Control and Prevention, Henan, China; 5 Queshan Center for Disease Control and Prevention, Henan, China; 6 Weishi Center for Disease Control and Prevention, Henan, China

## Abstract

Antiretroviral therapy has significantly expanded and an increased proportion of patients have switched to second-line regimens in China. We describe the outcomes of second-line therapy among patients having received long-term first-line ART. A prospective follow-up study was conducted in rural areas in China. We compared the virological, immunological outcomes and genotypic drug resistance (DR) profiles before and after regimen switches. A total of 303 patients were enrolled, 283 (93.4%) were retained at 12 months. Of 90 participants with HIV-RNA ≥ 1000 copies/ml before switch, the proportion of viral load (VL) ≥ 1000 copies/ml at 6 and 12 months was 49.4% and 43.9%, respectively. Of 213 patients with HIV-RNA < 1000 copies/ml before switch, the proportion of VL ≥ 1000 copies/ml at 6 and 12 months was 4.8% and 6.5%, respectively. The rates of drug resistance to NNRTIs, NRTIs, PIs decreased from 65.5%, 53.3%, and 1.1% before regimen switch to 26.8%, 18.3%, and 0% at 12 months, respectively. DDI-based initial ART regimens and missing doses in past month were associated with HIV RNA ≥ 1000 copies/ml at 12 months. The results showed that patients having received long-term first-line ART and experiencing virological failure had good virological outcomes after switching to second-line treatment in China.

The rapid scale-up of combination antiretroviral treatment (cART) over the past decade has resulted in dramatic reductions in morbidity and mortality for patients infected with HIV/AIDS^1^,^2^,^3^,^4^,^5^. The World Health Organization (WHO) estimated that at the end of 2011, more than 8 million HIV infected people were receiving antiretroviral therapy (ART) in low- and middle-income countries, up from 6.6 million in 2010 and representing an increase of about 20%^6^. However, the international failure rate of NNRTI-based treatment was 4.9% (95% CI, 3.9%–6.1%) at week 48^7^. A number of patients can be expected to develop drug resistance to first-line regimens, and a growing number of patients on ART in developing countries have switched to second-line therapy^8^,^9^,^10^. The WHO ever predicted that between 500,000 and 800,000 HIV-infected people on first-line combination ART would require switching to second-line therapy by 2010^11^.

After a pilot cART program among former plasma donors in 2002, China has scaled up the National Free Antiretroviral Treatment Program (NFATP) rapidly since 2003. Enormous efforts have been made to develop the capability of treating large numbers of people across wide geographic areas, including training rural healthcare workers. According to the NFATP guidelines, the baseline evaluation including CD4 cell count, complete blood count, transaminase levels, TB test, and serologic test for HBV and HCV should be performed before the initiation of combined antiretroviral therapy (cART). Since 2003, patients started cART when the CD4 cell counting was below 200 cells/mm^3^, and this CD4 threshold increased to 350 cells/mm^3^ in 2012. The threshold CD4 cell count further increased to 500 cells/mm^3^ in 2014, and the role of ART in public health programs was strengthened in the treatment and prevention of HIV infections, in accordance with the WHO 2013 consolidated guidelines. Besides CD4 counts, disease progression, and the status of TB, HBV and HCV coinfections are also taken into account to decide when to start cART. ART Initiation is recommended in HIV-infected individuals who are pregnant women, in HIV-serodiscordant couples, in those with active TB disease, or with severe chronic liver disease, regardless of CD4 cell count. During ART, CD4 cell count testing is done every 6 month, and plasma viral load is quantified at least once a year. In addition, hepatic function is monitored at months 0, 0.5, 1, 2, 3, 6 of cART, and every 6 months thereafter. The first-line regimens are composed of two NRTIs and one NNRTI, which were ddI-based as AZT/d4T + ddI + NVP/EFV before 2005, and 3TC-based as AZT/d4T + 3TC + NVP/EFV since 2005, and AZT/TDF + 3TC + EFV/NVP since 2012. In case of HIV/HCV coinfection, treatment decision is made based on CD4 cell count and hepatic function. Initiation of ART is recommended prior to starting the treatment for HCV when CD4 cell count is below 200 cells/mm^3^, and HCV treatment is recommended before the initiation of ART when CD4 cell count is ≥350 cells/mm^3^, or when CD4 cell count is 200 to 350 cells/mm^3^ and with elevated liver enzymes. In addition, antiretroviral drugs with less hepatoxicity should be chosen, and the recommended regimen is TDF + 3TC + EFV/(LPV/r)^12^.

By the end of January 2014, more than 281, 873 patients across the country have received free antiretroviral treatment^13^. Many studies have found that the NFATP has decreased mortality among Chinese HIV patients^12^,^14^,^15^,^16^. Some patients entering NFATP were former plasma donors who were infected by HIV through unhygienic plasmapheresis procedures in the mid-1990s in rural central China^17^. With increasing time on treatment, some patients are likely to experience drug resistance, virological failure, or immunological failure. A study in South Africa reported that patients who remained on failing first-line therapy had a higher risk of mortality within a year than those who switched to second-line drugs^18^. The aim of this prospective observational survey is to evaluate the outcomes of second-line therapy and drug resistance profiles among patients receiving long-term first-line therapy in rural areas of China.

## Results

### Demographic Characteristics

Among 320 consecutive patients enrolled, 17 patients were excluded from this study, including two who were less than 18 years old, ten who received first-line treatment for less than 12 months, and five patients who did not switch to second-line antiretroviral drugs. Totally, 303 subjects entered this prospective cohort. Blood samples for testing viral load and drug resistance were collected from all patients within a month before switching drugs (baseline). The characteristics of these patients at baseline are shown in Table 1. The mean age was 48.0 years (SD, 8.6 years), 61.1% were female, 87.8% married, 93.7% were infected through blood donation, 85.1% were HCV antibody test positive, and the median duration of first-line ART was 89 months. After 12 months of regimen switch, 283 (93.4%) participants were still on second-line ART (Table 1).

### Responses to Second-line Regimen

Among the 303 participants, 90 participants had plasma HIV viral load ≥1000 copies/ml and 213 patients with viral load <1000 copies/ml at baseline (Table 2). Of the former 90 participants, the rate of HIV/HCV coinfection was 81.1% (73/90), the median CD4 counts before regimen switch, 6 and 12 months after switching were 266, 275, and 299, respectively, and the proportion of VF after switching to second-line for 6, and 12 months was 49.4%, 43.9%, respectively (χ^2^ = 61.09, p < 0.0001). It was notable that a substantial proportion of patients were with VF but not detectable drug resistance at 6 months (47.5%, 19/40) and 12 months (36.1%, 13/36) of regimen switch. For those with HIV-RNA <1000 copies/ml at baseline, the rate of HIV/HCV coinfection was 86.8% (185/213), and the proportion of patients with VF was 4.8%, 6.5% at 6 and 12 months of switching to second-line regimen, respectively (χ^2^ = 12.33, p = 0.0004). Among those patients with viral load ≥1000 copies/ml at 6 and 12 month, 60.0% (6/10) and 69.2% (9/13) had not detectable drug resistance, respectively. The patients with VF at switch had much higher rates of drug resistance at 6 and 12 months than those patients with HIV RNA < 1000 copies/ml at baseline (p < 0.001 at 6 months, p < 0.001 at 12 months), and there was also significant difference in the proportion of VF after switching to second-line at 6 and 12 months between these two groups (p < 0.001 at 6 months, p < 0.001 at 12 months).

Among 60 patients with drug resistance at baseline (Table 3), the proportion of viral load ≥1000 copies/ml at baseline and after switching to second-line for 6, and 12 months was 100%, 48.1%, 41.8%, respectively (χ^2^ = 43.12, p < 0.0001). Twenty-eight patients had constant VF at baseline and after 6 and 12 months of regimen switch. Disregard of the existence of drug resistance at switch, the rates of VF was dramatically decreased among the patients with baseline VF after 6 and 12 months (drug resistance vs. without drug resistance at baseline, p = 0.75 at 6 months, p = 0.59 at 12 months).

Among the 213 patients with viral load <1000 copies/ml before switching regimens, 24 (11.3%) had CD4 < 200 (Table 4). Among these patients, the proportion with viral load ≥1000 copies/ml 6 and 12 months after switching to second-line was 21.7% and 21.0%, respectively. Among the 189 patients with viral load <1000 copies/ml and CD4 ≥ 200, the proportion with viral load ≥1000 copies/ml 6 and 12 months after switching regimens was 2.7% and 4.9%, respectively. The difference in the proportion of VL < 1000 copies/ml at 6 and 12 month was significant (p = 0.02 at 6 months, p < 0.01 at 12 months, respectively) between these two groups.

### Genotypic Drug Resistance Profile and Risk Factors for Failure of Second-line ART

**Figure 1:**
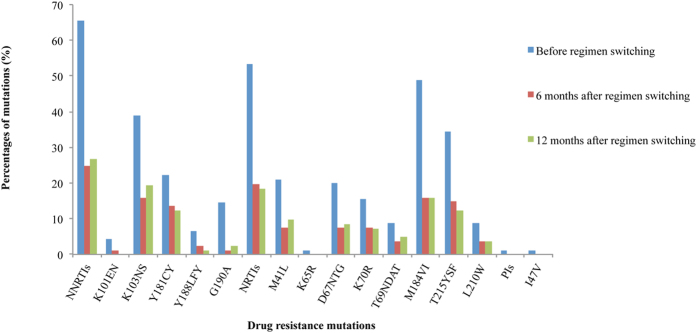
HIV drug resistance mutations among patients with HIVDR mutations detected before regimen switching, 6 and 12 months after regimen switching.

Ninety samples from participants with VF at baseline were genotyped with partial *pol* region. Based on phylogenetic analyses, two (2.2%) of them harbored B/C unique recombinant forms (URFs) HIV-1 strains; others had Thai B (B’) viruses. At baseline, 60 (66.7%) patients had drug resistance, and the proportion of drug resistance to NNRTIs, NRTIs, and PIs were 65.5%, 53.3%, and 1.1%, respectively. Commonly observed NRTI-related mutations were 3TC related M184V mutation (48.9%), Thymidine analogue such as AZT/d4T associated mutations (TAMs) like T215YSF (34.4%) and M41L (21.1%), non-thymidine analogue such as DDI related K65R mutation (1.1%), and L74V (1.1%). 31.1% of the patients had at least 2 TAMs. The most frequent NNRTI-related mutations included K103NS (38.9%), Y181CY (22.2%), G190A (14.4%). A PI-related mutation, I54V, was found in one patient. One of the two patients with B/C URF harbored multiple DR mutations, including NRTI-related mutations as D67N, K70R, M184V, and K219E, which conferring high resistance to 3TC, and low resistance to TDF, and NNRTI-related mutations as E138Q, V179D, and G190Q, causing high resistance to all current available NNRTIs. One year after switching to second-line ART among the same individual, 36 (43.9%) patients had plasma HIV RNA ≥ 1000 copies/ml, and the proportion of drug resistance to NNRTIs, NRTIs, and PIs were 26.8%, 18.3%, and 0%, respectively. The proportion of patients having at least 2 TAMs was 17.3%, 13.4%, respectively, at 6 and 12 months. Of the 28 patients who were with constant VF, and were mentioned above, two patients did not harbor drug resistance mutants at baseline but developed drug resistance at 12 month after the switch to the second line drugs, 11 patients did not have drug resistance at all the three time points, and the remaining 15 patients had detectable drug resistance at the three time points, five of these 15 patients have at least one more TAMs mutation, and one of the 15 patients developed a 3TC related M184V mutation. The patient with drug resistance B/C URF was lost of follow up at 6 months, and had plasma HIV RNA > 1000, but without drug resistance at 12 months. The other patient with B/C URF without detectable drug rsistance at baseline achieved viral suppression at both 6 and 12 months. Figure 1 shows the prevalence of HIV drug resistance mutations before and after switching drugs at 6 and 12 month. Details are shown in Supplementary Table s1.

Factors significantly associated with HIV RNA ≥ 1000 copies/ml after switching to second-line ART at 12 months are shown in Table 5. DDI-based initial ART regimen, having missed doses in the past month, and lack of social and emotional supports were risk factors associated with virological failure (HIV RNA ≥ 1000 copies/ml). The rates of missing doses in the past month among 30 patients with baseline VF and without detectable resistance mutations were 36.7% at baseline, and 48.2% after 12 months. Among patients with baseline VF, no significant difference was found in adherence between persons with and without detectable DR mutations (p = 0.13 at baseline, p = 0.65 at 12 months, respectively). In the multivariable logistic regression model, DDI-based initial ART regimen [adjusted odds ratio (AOR) = 3.45, 95% CI: 1.08, 10.98; P = 0.036] and missed doses in past month (AOR = 4.61, 95% CI: 1.58, 13.40; P = 0.005) were associated with virological failure.

## Discussion

In this one-year prospective cohort, we found that among 90 patients who switched to a second-line regimen with viral load ≥1000 copies/ml, 43.9% had HIV RNA ≥ 1000 copies/ml 12 months later. The rate of virological failure in this study was substantially higher than what was reported in other low-resource settings^19^,^20^,^21^. Kim and colleagues found the rate of virological failure in South-Africa was 13.9% at 12 months after switching; Siripassorn and colleagues found the overall virological failure rate at 24 months was 15.2% in a resource-limited setting. In a systematic review of 19 low-income countries including 2035 patients, Ajose and colleagues found that the cumulative pooled proportion of adult patients failing virologically after switching to second-line for 6 and 12 months was 21.8% and 23.1%, respectively. Patients in our study had received first-line treatment for a long time, and the median duration was 89 months. Previous studies have shown that longer duration of initial treatment was associated with more low-level drug resistance mutations^22^, and increased the complexity of resistance genotypes^23^,^24^,^25^,^26^, thereby reduced drug sensitivity. Therefore, it is possible that the evolution of drug-resistant strains under diverse drug selective pressure was different from that in other studies. In addition, lack of adherence data makes it hard to compare our results with other studies in resource-limited settings.

The patients in this study were the first groups of patients receiving the first line cART drugs in China. Among the patients with VL < 1000 copies/mL who switched to the second-line regimens, 86.9% (185/213) were HCV antibody positive. In case of HIV/HCV co-infection, TDF + 3TC + EFV/(LPV/r) is recommended by the NFATP guidelines. Even though all the patients had an HCV antibody test, the second-line drugs had been unavailable for many years after the initiation of their cART. Among the 185 patients, 56.8% had received DDI-based regimen as their initial treatment, and 41.6% had received AZT-based or D4T-based regimen. The other 28 patients demanded to switch to second-line regimen, because they believed that the second-line regimens could better control the virus and have fewer side effects. We found that a small proportion of the patients with VL < 1000 copies/ml at regimen switch, had viral load rebounded to above 1000 copies/ml. However, it’s notable patient with lower CD4 counts seems more likely to experience virological failure after switching. Comparing two groups with different viral load levels before switching, Patients with viral load <1000 at regimen switch had much lower proportions of virological failure (viral load ≥1000 copies/ml) and drug resistance at 6 and 12 months of regimen switch than other patients with viral load ≥1000 at switch. However, the long-term benefit of earlier second-line switch is still not clear. More studies are needed to clarify the optimal switch criteria.

In this study, the majority of the patients who were former plasma donors (FPD), with VF at baseline were infected with B’ HIV-1 strains. The distribution of HIV-1 subtypes among FPD is consistent with the findings found in a cross-sectional national molecular survey^27^,^28^. As the number of patients with VF at baseline who were infected with HIV-1 strains other than B’ was limited, it had not enough power to compare the difference of virological and drug resistance outcomes between various HIV-1 subtypes.

Among 90 patients with virological failure before treatment switch, the rate of drug resistance to NNRTIs, NRTIs, PIs was 65.5%, 53.3%, 1.1%, respectively, and the multi-drug resistance to both NNRTIs and NRTIs was 52.2%. After switching regimens for 12 months, 22 cases (26.8%) displayed NNRTI mutations and 15 cases (18.3%) displayed NRTI mutations. Although one patient harbored PI-related I54IV mutation before switching, no PI-related mutation was identified after 12 months. As this patient did not have any experiences of treatment with PIs before switching regimen, the I54V mutation might occur naturally as a polymorphism, and disappeared after the second-line treatment. Of 60 participants with drug resistance before switching regimens, the proportion with viral load >1000 copies/ml before and after switching to second-line for 6, and 12 months was 100%, 48.1%, 41.8%, respectively, of 30 patients without drug resistance the proportion was 100%, 51.8%, 48.1%, respectively. Although these two groups had experience virological failure before switching drugs, the rate of viral suppression both decreased, and there was no statistical difference. Despite of drug resistance at baseline, the rate of drug resistance decreased and the proportion of VL < 1000 copies/ml increased after switching to second-line regimens. Our study showed that the switch to second-line therapy in patients with long-term treatment and drug resistance led to effective viral suppression again, although drug resistance to NRTIs widely exists, especially drug resistance mutations such as M41L, T215Y, and TAMs, has a great influence on TDF sensitivity in second-line drugs^29^. Our study demonstrates that prescribed PI-based regimens can successfully re-suppress the replication of NRTI- and NNRTI- resistant HIV mutants, improve virological and immunological effect, even in the absence of a fully active NRTI backbone. This finding is similar to that of other studies^20^,^30^. But some other studies had shown that the accumulation of drug resistance mutation was harmful for the effectiveness of subsequent drugs, especially NRTIs backbone treatment^31^. Therefore it is necessary to further assess the impact of the cumulative drug resistance mutations on the second-line drug treatment. Although NNRTIs drugs were replaced by PIs, some NNRTI-related resistance mutations such as K103N, Y181C, Y188L and G190A were still detected in some patients after 12 months of regimen, which confirmed that these two mutation sites maybe have little impact on virus replication ability1^32^,^33^. It’s notable that the latter three mutations reduce susceptibility not only to the first generation NNRTIs such as NVP and EFV, but to the second generation NNRTIs such as etravirine (ETR) and rilpivirine (RPV).

Our study found that DDI-based initial ART regimen, and missed doses in the month prior to switching were independently associated with virological failure (HIV RNA ≥ 1000 copies/ml) 12 months after switching to second-line ART in China. Adherence remains as a significant risk factor affecting viral suppression, and these results are similar to those obtained in other developing countries^34^. One study found that patients with a self-reported adherence of less than 80% were 3.14 times more likely to experience virological failure than patients with self-reported adherence of at least 95%^30^. Another study found that missed doses were associated with virological failure^31^. Virological failure as a result of poor adherence reminds us of the importance of adherence in all settings in which ART is administrated, and indicates that strengthening education and counseling among HIV patients should be a priority for HIVDR prevention in China^35^,^36^. Improved training for health care providers is also important, especially among those with limited resources to new treatment technologies.

This cohort study had several limitations. First, only 90 patients (29.7%) experienced virological failure before regimen switch, so conclusions should be drawn with caution. Second, due to the limited geographic distribution of the surveillance sites and exclusion criteria, these results may not be fully representative of HIV/AIDS patients in China. More data are needed, particularly for patients receiving long-term treatment, to better understand outcomes after switching regimens. Third, due to the small sample size, the HIV cumulative drug resistance rate after receiving first-line therapy for a median of 89 months is likely lower than the true drug resistance rate. Finally, an one-year follow up survey is relatively short for the purposes of assessing outcomes after switching to second-line therapy for patients receiving long-term first-line treatment, and more studies with long-term monitoring are recommended to provide more accurate data on viral suppression, and HIVDR profiles.

In summary, this 12-month prospective study demonstrates good virological outcomes after switching to second-line treatment for patients having received long-term first-line treatment and experiencing virological failure at the time of switching regimens. However, for patients with viral load <1000 copies/ml, switching to second-line treatment is not clearly beneficial, and more studies are needed to clarify the optimal switch criteria. The poor adherence is associated with an increased risk for poor virological response.

## Methods

### Study design and study participants

Three sentinel surveys were conducted in rural areas of China, including Weishi county, Xincai county and Queshan county of Henan province, where ART began earlier and there were a large of patients having received long-term first-line regimen. These areas have high rates of HIV drug resistance (HIVDR). The study was a prospective cohort study with follow-up at 6 and 12 months after switching to second-line regimens. Study subjects were recruited at the time of switching to second-line therapy at each clinic. Criteria for enrolling in the study were: previously receiving first-line therapy for at least 12 month, 18 years or older, having national ART patient ID, switching to second-line regimen, and willing to provide informed consent. A baseline screening survey was conducted between April 2012 and November 2012 before switching antiretroviral drugs. ART regimens were provided through the NFATP. Second-line ART regimens consisted of tenofovir (TDF) + lamivudine (3TC) + lopinavir and ritonavir (LPV/r). Based on WHO guideline, we defined viral load ≥1000 copies/ml as virological failure. The main study outcome was HIVDR variation after switching regimens. Specific objectives were to: (1) estimate changes in the proportion experiencing virological failure, drug resistance and immunological failure after switching second-line ART; (2) study factors associated with virological failure after switching second-line ART at 12 months.

### Ethics Statement

The study was approved by the institutional review board (IRB) at the National Center for AIDS/STD Control and Prevention of the China Center for Disease Control and Prevention (NCAIDS, China CDC). All experimental protocols were approved by IRB at NCAIDS, China CDC, according to the international and Chinese ethical guidelines, and the methods were carried out in accordance with the approved guidelines.

### Data collection

Data were collected using an interviewer-administered questionnaire. The questionnaire was administered by trained interviewers in a local clinic room. Questionnaires included demographic data, ART treatment and self-reported adherence data. Demographic variables included age, sex, marital status and HIV transmission route. ART treatment variables included initial regimens, duration of first-line treatment. Self-reported adherence variables included emotional support, missed ART doses in the past month, the ratio of on-time drug intake in the past month, and any regimen terminations in the past month.

### Laboratory tests

All subjects provided blood specimens before switching drugs and at 6 and 12 months after switching to measure CD4+ T-lymphocyte count (CD4 count), HIV viral load, and HIV drug resistance mutations. HCV antibody test just done before switching drugs. CD4 count was measured using flow cytometry (FACSC Calibur, BD Company, USA) within 24 hours after specimen collection in local CDC facilities. Plasma was isolated by centrifuge and stored frozen at −80 °C at local CDC facilities before transferring to NCAIDS in Beijing for measuring viral load and drug resistance mutations. Plasma HIV RNA was quantified with real-time NASBA (NucliSense Easy Q, bioMérieux, France) according to the manufacturers’ recommendations. Successful viral suppression was defined as HIV RNA level < 1000 copies/ml. If viral load ≥1000 copies/ml, HIVDR genotyping was performed by an in-house polymerase chain reaction at the WHO Accreditation Laboratory of NCAIDS, China CDC^37^,^38^. A 1.3 kb fragment of the HIV pol gene (protease 1–99 amino acids and part of reverse transcriptase 1–250 amino acids) was amplified for drug resistance mutation analysis and viral subtype determination. Successfully amplified sequences were analyzed for HIVDR using the Stanford University HIV Drug Resistance Database online sequence analysis tool (http://hivdb.stanford.edu/pages/algs/sierra_sequence.html). Any drug resistance mutations, whether conferred low-, intermediate-, or high-level, were defined HIVDR mutations^39^,^40^,^41^.

### Data analysis

EpiData software (The EpiData Association Odense, Denmark) was used for data entry. Data were double-blinded entered and compared. Questionnaire and laboratory data were analyzed using Statistical Analysis System version 9.1 (SAS Institute Inc., Cary, NC, USA). Unadjusted odds ratios were computed and tests for significance were based on chi-square and Fisher’s exact tests. Variables that were significantly (p < 0.05) associated with HIV RNA ≥ 1000 copies/ml at 12 months in the univariable analyses were considered for inclusion in the multivariable logistic regression model. Multivariable logistic regression was performed to examine the independent effect of each factor under consideration. P-values < 0.05 were considered statistically significant, and all tests of significance were two-sided.

## Additional Information

**How to cite this article**: Wang, J. *et al.* Efficacy and HIV drug resistance profile of second-line ART among patients having received long-term first-line regimens in rural China. *Sci. Rep.* **5**, 14823; doi: 10.1038/srep14823 (2015).

## Supplementary Material

Supplementary Information

## Figures and Tables

**Table 1:** Characteristics of HIV patients ART at baseline and the rates of retention at 6 and 12 months of regimen switching.

Characteristics	N(%)
Overall	303
Age (mean ± SD, year)	48.0 ± 8.6
Sex	
Male	118(38.9)
Female	185(61.1)
Marital status	
Married	266(87.8)
Other	37(12.2)
Possible HIV transmission route	
Blood donation	284(93.7)
Sexual contact	16(5.3)
Others	3(1.0)
HCV antibody test	
Negative	45(14.9)
Positive	258(85.1)
Initial ART regimen*	
DDI + AZT/D4T + NVP	175(57.7)
AZT + 3TC + NVP/EFV	62(20.5)
D4T + 3TC + NVP/EFV	55(18.1)
other	11(3.6)
Duration of receiving first-line ART regimen (median, month)*	89
CD4 count (cell/mm^3^) before switching to the second-line ART	
200 or above	242(80.4)
0–199	59(19.6)
HIV RNA ≥ 1000 copies/ml before switching to the second-line ART	90(29.7)
Reasons for not retained at 6 months	
Death	5(1.6)
Loss to follow-up	9(3.0)
Reasons for not retained at 12 months	
Death	4(1.3)
Loss to follow-up	11(5.0)
Retention at 12-month follow-up study	283(93.4)
Duration of follow-up (median, month)	10.0

Note: *variables related the initiate regimen; all other variables were collected before the patients switching drugs.

**Table 2:** CD4 count, viral load and HIV drug resistance at months 6 and 12 of switching regimen among patients with VL≥ or <1000 copies/ml at baseline.

Characteristics	No. of patients	Before regimen switch				6 month after regimen switch			12 month after regimen switch		
		HCV antibody test PositiveN(%)	Median CD4 count (cell/mm^3^)	VL ≥ 1000 N(%)	HIVDR N(%)	Median CD4 count (cell/mm^3^)	VL ≥ 1000 N(%)	HIVDR N(%)	Median CD4 count (cell/mm^3^)	VL ≥ 1000 N(%)	HIVDR N(%)
Total	303	258(85.1)	352	90(29.7)	60(19.8)	390	50(17.3)	25(8.6)	395	49(17.3)	27(9.5)
At baseline											
VL ≥ 1000	90	73(81.1)	266	90(100.0)	60(66.7)	275	40(49.4)	21(25.9)	299	36(43.9)	23(28.0)
VL < 1000	213	185(86.8)	391	0(0)	0(0)	434	10(4.8)	4(1.9)	424	13(6.5)	4(2.0)

**Table 3:** CD4 count, viral load and HIV drug resistance at months 6 and 12 of switching regimen among patients with VL ≥ 1000 copies/ml at baseline (n = 90).

Characteristics at baseline	No. of patients	Before regimen switch			6 month after regimen switch			12 month after regimen switch		
		Median CD4 count (cell/mm^3^)	VL ≥ 1000 N(%)	HIVDR N(%)	Median CD4 count (cell/mm^3^)	VL ≥ 1000 N(%)	HIVDR N(%)	Median CD4 count (cell/mm^3^)	VL ≥ 1000 N(%)	HIVDR N(%)
VL ≥ 1000										
With DR	60	245	60(100)	60(100)	264	26(48.1)	20(37.0)	279	23(41.8)	21(38.2)
Without DR	30	364	30(100)	0(0)	281	14(51.8)	1(3.7)	384	13(48.1)	2(7.4)

**Table 4:** CD4 count, viral load and HIV drug resistance at months 6 and 12 of switching regimen among patients with VL < 1000 copies/ml at baseline (n = 213).

Characteristics at baseline	No. of patients	Before regimen switch			6 month after regimen switch			12 month after regimen switch		
		Median CD4 count (cell/mm^3^)	VL ≥ 1000 N(%)	HIVDR N(%)	Median CD4 count (cell/mm^3^)	VL ≥ 1000 N(%)	HIVDR N(%)	Median CD4 count (cell/mm^3^)	VL ≥ 1000 N(%)	HIVDR N(%)
VL < 1000 and										
CD4 < 200	24	132	0(0)	0(0)	190	5(21.7)	2(8.7)	242	4(21.0)	1(5.3)
CD4 ≥ 200	189	405	0(0)	0(0)	443	5(2.7)	2(1.1)	451	9(4.9)	3(1.6)

**Table 5:** Factors associated with HIV RNA ≥ 1000 copies/ml after switching to second-line therapy for 12 months among patients with viral load >1000 copies/ml at baseline.

Variable	Number	HIV RNA ≥ 1000 copies/mlN(%)	Crude OR(95% CI)	*P-value*	Adjusted OR(95% CI)	*P-value*
Total	82	36(43.9)				
Age (year)						
≤50	23	14(60.9)				
>50	59	22(37.3)	0.38(0.14, 1.03)	0.0569		
Sex						
Female	46	18(39.1)				
Male	36	18(50.0)	1.56(0.64, 3.78)	0.3260		
Marital status						
Other	10	4(40.0)				
Married	72	32(44.4)	1.20(0.31, 4.62)	0.7909		
HIV transmission route						
Other	7	4(57.1)				
Blood donation	75	32(42.7)	0.56(0.12, 2.67)	0.4653		
HCV antibody test						
Negative	15	5(33.3)				
Positive	67	31(46.3)	1.72(0.53, 5.58)	0.3650		
DDI-based initial ART regimen						
No	25	8(32.0)				
Yes	57	28(49.1)	2.05(0.76, 5.51)	0.1539	3.45(1.08, 10.98)	0.0362
Drug resistance before switching to second ART						
No	27	13(48.1)				
Yes	55	23(41.8)	0.77(0.31, 1.95)	0.5876		
Stop the regimen in the past month						
No	78	33(42.3)				
Yes	4	3(75.0)	4.09(0.41, 41.10)	0.2314		
Missed doses in the past month						
No	52	16(30.8)				
Yes	30	20(66.7)	4.50(1.72, 11.76)	0.0022	4.61(1.58, 13.4)	0.0051
Always/often get social and emotional support						
No	33	21(63.6)				
Yes	49	15(30.6)	0.25(0.01, 0.64)	0.0038		
