# Supplementary material for: Efficacy and HIV drug resistance profile of second-line ART among patients having received long-term first-line regimens in rural China
Source: Sci Rep. 2015 Oct 8;5:14823. doi: 10.1038/srep14823 (PMC4597210; doi:10.1038/srep14823)
Supplement: Supplementary Information [file srep14823-s1.pdf]

Efficacy and HIV drug resistance profile of second-line ART among patients receiving long-term first-line regimens in rural China

Jing Wang[1,2#], Zhe Wang[3#], Jia Liu[3#], Yanchao Yue[4], Shimei Yang[5], Huimin Huang[6], Cui He[1], Lingjie Liao[1], Hui Xing[1*], Yuhua Ruan[1], Yiming Shao[1*]

Supplementary Table S1. Prevalence of HIV drug resistance mutations before and at 6 and 12 months after switching drugs

	Before regimen switch N=90 (%)	6 month after regimen switch N=81 (%)	12 month after regimen switch N=82 (%)
NNRTIs	59(65.5)	20(24.7)	22(26.8)
K101EN	4(4.4)	1(1.2）	0
K103NS	35(38.9)	13(16.0)	16(19.5)
Y181CY	20(22.2)	11(13.6）	10(12.2)
Y188LFY	6(6.7)	2(2.5)	1(1.2)
G190A	13(14.4)	1(1.2)	2(2.4)
NRTIs	48(53.3)	16(19.7)	15(18.3)
M41L	19(21.1)	6(7.4)	8(9.7)
K65R	1(1.1)	0	0
L74V	1(1.1)	1(1.2)	1(1.2)
D67NTG	18(20.0)	6(7.4)	7(8.5)
K70R	14(15.5)	6(7.4)	6(7.3)
T69NDAT	8(8.9)	3(3.7)	4(4.9)
M184VI	44(48.9)	13(16.0)	13(15.8)
T215YSF	31(34.4)	12(14.8)	10(12.2)
L210W	8(8.9)	3(3.7)	3(3.6)
PIs	1(1.1)	0	0
I47V	1(1.1)	0	0
